# Do We Need to Worry About *Staphylococcus epidermidis* ST0409KOC, a Cheese‐Isolated Strain With Bacteriocinogenic Properties?

**DOI:** 10.1002/mnfr.70359

**Published:** 2026-01-15

**Authors:** Kayque Ordonho Carneiro, João Marcos Scafuro Lima, Svetoslav Dimitrov Todorov

**Affiliations:** ^1^ ProBacLab, Laboratório de Microbiologia de Alimentos Departamento De Alimentos e Nutrição Experimental Food Research Center Faculdade De Ciências Farmacêuticas, Universidade de São Paulo São Paulo São Paulo Brazil; ^2^ CISAS ‐ Center for Research and Development in Agrifood Systems and Sustainability Instituto Politécnico de Viana do Castelo Viana do Castelo Portugal

**Keywords:** antimicrobial, bacteriocins, cheese, *Staphylococcus epidermidis*, virulence genes

## Abstract

*Staphylococcus epidermidis* is widely found throughout the entire length of human skin and animals since the first year of life, providing a natural protective barrier against other pathogenic microorganisms. Therefore, related to its reputation, it is of utmost importance to investigate the virulence capacity for strains of this species. The aim of this study was to evaluate the safety of *S. epidermidis* ST0409KOC, a strain isolated from Bulgarian feta‐type cheese, and to explore its bacteriocinogenic property against different strains of *Listeria monocytogenes* and other pathogens. *S. epidermidis* ST0409KOC can be considered a safe strain regarding its virulence genetic background, presenting only a virulence gene of *IS257* among the 18 genes investigated. Moreover, it showed high bacteriocinogenic activity against different serotypes of *L. monocytogenes*, and its bacteriocin may be of great interest for controlling this pathogen. However, bacteriocin production when associated with pathogenic or opportunistic strains can be considered as a virulence factor, since it will improve survival abilities for the producer. Related to the observed results regarding the safety of the investigated strain, production of bacteriocin, and potential beneficial features, the *S. epidermidis* ST0409KOC strain is a strong candidate for use as a probiotic in different applications, and further studies are needed.

## Graphical abstract

Bacteriocinogenic *Staphylococcus epidermidis* ST0409KOC was isolated from cheese and characterized as bacteriocin‐producing with strong activity versus *Listeria monocytogenes* and other pathogens. *S. epidermidis* ST0409KOC can be considered a safe strain regarding its virulence genetic background, presenting only a single virulence gene of *IS257* among the 18 genes investigated.

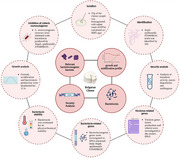

## 1 Introduction

Bacterial species are natural inhabitants of distinct biotic and abiotic ecological niches and contribute to the digestion processes in the gastrointestinal tract, are actively involved in modulation of the immune system and serve as essential barrier in humans and other animals, moderate the fermentation processes in food formulation, or contribute to the technological development in the biotechnological and pharmaceutical industry amid the production of different beneficial metabolites. However, some microbial cultures, associated with their metabolisms can be considered pathogenic and/or opportunistic and are of great concern in the cause of foodborne diseases (FBDs), nosocomial infections and bacteria multi‐resistant to conventional antimicrobial therapies [1, 2, 3].

*Staphylococcus epidermidis* is a Gram‐positive, non‐motile bacterium that belongs to the genus *Staphylococcus* [1]. The genus *Staphylococcus* is well known for harboring several pathogenic species, such as *Staphylococcus aureus*, a highly virulent pathogen, possessing the coagulase enzyme, this ability being used to differentiate members of the genus *Staphylococcus* [2]. *S. epidermidis* is a coagulase‐negative *Staphylococcus* (CoNS), a small group within its genus, where it is widely found throughout the entire length of human skin, with moist areas being its most prevalent, for example, mucous membranes [3].

Based on the data from the metagenomic analyses, it was pointed out that several CoNS strains may inhabit human skin and mucous membranes, with *S. epidermidis* being the most abundant species, adapted to the high saline concentrations and low pH, and nutrients present in these regions of humans and other animals. *S. epidermidis* is naturally associated with humans and other animals since the first year of life, composing epithelial microbiota, providing a natural protective barrier against other pathogenic microorganisms [4].

Its presence in the largest human organ, the skin, is possible due to its resistance to the conditions present there and the presence of adhesins on its surface, which provide the ability to adhere to the skin [5]. Furthermore, due to the circumstances mentioned above, *S*. *epidermidis* is able to participate in the maturation of the immune system by inducing the chemokine CCL20, recruiting Treg immune cells [4], which in turn are responsible for skin homeostasis [6].

With the objective to survive, in evolutionary processes, some bacterial cultures have developed the ability to form biofilm, a matrix composed of a cluster of microorganisms, water, and extracellular polymers (extracellular polymeric substance—EPS). As a consequence, the biofilm complex allows the inhabitant bacteria to “take shelter” in this protective barrier, protecting them from stressful environmental conditions such as the effect of antibiotics and other antimicrobials, sanitizers, radiation, and simple conventional cleaning processes with chemical, physical, and thermal agents, making its removal a difficult factor to perform [7]. Being a protective tool for bacterial cultures, biofilm formation properties are a major concern in the food industry, as biofilm formation abilities allow these bacteria to settle on food production surfaces, such as countertops, machines, cutting equipment, and even on water outlets such as spouts. As a result, biofilm formation may contribute to the stability and spread of microbial cultures related to FBDs or even food deterioration [7, 8]. *Listeria monocytogenes* is an undeniably recognized example of forming biofilm and contaminating the production plants, and its removal is resistant to conventional sanitizing treatments [9].

Moreover, some strains of *S. epidermidis* can be considered opportunistic bacteria because they can be associated with nosocomial infections, due to their high capacity to form biofilm [3] and establish themselves in abiotic environments [10]. There is a high prevalence of *S. epidermidis* in nosocomial infections, and CoNS are known to be responsible for infections of central and peripheral venous catheters, causing great concern to health professionals, where approximately 180 million peripheral intravascular catheters and 7 million central venous catheters are used annually in the United States, totaling more than two billion dollars, due to the high need for frequent replacement [1, 10]. Not only that, 20% of the genes of *S. epidermidis* are variable, that is, this species has a high capacity for genetic transfer between other strains [11]. The transfer of genetic elements, plasmids, and phages occurs horizontally between strains, making the capacity for increased virulence possible. Bacteria previously sensitive to certain antimicrobial therapies become resistant, or strains previously considered safe acquire the ability to produce different toxins [4].

Bacteriocins are one of the natural antimicrobial metabolites produced by different bacterial groups [12], show to be one of the essential players in the reduction of food borne contaminants [13] and even suggested as alternatives for the antibiotics in human and other animals clinical practice [14]. Moreover, bacteriocin production when associated with pathogenic strains, can be considered as a virulence factor, since it will improve survival abilities for the producer [15]. From the physiological point of view, microbial compounds, including bacteriocins, have the ability to penetrate this protective matrixes of the biofilms and to present specific antimicrobial action against pathogens involved in the formation of biofilms [7]. Data from Lima [7], Pérez‐Ibarreche et al. [16], and Fugaban et al. [17], show the ability of bacteriocins produced by lactic acid bacteria (LAB) to permeate the biofilm and inhibit or reduce its formation by pathogens, without interfering with the growth of planktonic cells for 48 h. Ohn et al. [3] found inhibitory properties of the *Escherichia coli* ATCC 35218 strain on the formation of *S. epidermidis* ATCC 35984 biofilm, as well as reducing the number of planktonic cells in its presence.

Bacteriocins have gained attention due to their wide use and different innovative applications. By nature, bacteriocins are ribosomal polypeptides produced with antimicrobial action [18]. However, some of them may be involved in microbial interactions, including *quorum sensing* [19]. For most of the bacteriocins was suggested that they can cause cell death of target cells by creating pores in the cell membrane or causing injury to the cell's natural cell cycle [20], or bacteriocinogenic strains can produce small amounts in order to prevent *quorum‐sensing* recognition between cells and inhibit the establishment of biofilms [19].

Therefore, the aim of this study was to evaluate the safety of *S. epidermidis* ST0409KOC, a strain isolated from Bulgarian feta‐type brined cheese, and to explore its bacteriocinogenic property against different strains of *L. monocytogenes* and other pathogens.

## 2 Materials and Methods

In the process screening of bacteriocinogenic strains, cheese samples from feta cheese from medium‐scale producers from Bulgaria were evaluated. The isolation process consisted of selecting microbial cultures with bacteriocinogenic properties according to the methodology described by Rwubuzizi et al. [21] with minor modifications. Cheese samples (25 g) were macerated with 225 mL of sterile saline solution (0.85% NaCl, *w/v*) for 5 min in a stomacher (Seward, West Sussex, UK) and serially diluted 10× with the sterile saline solution. In the following, 100 µL of each dilution was plated on MRS agar (Difco, Franklin Lakes, NJ, USA) and covered with an equal volume (*w/v*) of 2% agar (Difco). The plates were incubated for 24–48 h at 37°C, and the CFU/g were determined. Subsequently, plates that demonstrated well‐defined colonies were selected and covered with a layer of BHI (Difco), supplemented with 1% agar and *L. monocytogenes* ATCC 7644 to obtain final concentration of approximately between 10^5^ and 10^6^ CFU/mL, taking in consideration application of appropriate volume from the 24 h culture of applied test microorganism into the growth media destinated to be added as additional layer [7, 12]. The plates were then incubated for an additional 24 h at 37°C, followed by observation of the presence of inhibition zones around bacterial colonies. Colonies containing well‐defined inhibition zones (translucent zones) were considered to have anti‐*Listeria* action and selected to be cultured in MRS broth (Difco) for 24 h at 37°C, subjected to morphological analysis, Gram staining and catalase reaction as preliminary identification tests and further appropriate biochemical and physiological test as suggested by de Vos et al. [22] were performed, followed by storage in cryogenic tubes containing 20% glycerol (*v/v*) final concentration and maintained at −20°C for later analyses.

### 2.1 Inhibition Spectrum

The isolates were then subjected to bacteriocin production analysis according to the recommendations of Valledor et al. [23] with minimal modifications. The isolates were activated in MRS broth (Difco) for 24 h at 37°C, then centrifuged (10 000 ×*g*, 5 min, 4°C), pH adjusted to the value between 5.5 and 6.5 using sterile 3 M NaOH, and filtered in syringes with a 0.22 µm filter (Kasvi, São José dos Pinhais, SP, Brazil) and transferred to a sterile 1.5 mL Eppendorf. These aliquots were treated for 10 min at 80°C to reduce the potential effects of proteases or $H_{2}O_{2}$. Then, 10 µL of each treated aliquot was distributed by the spot‐on‐the‐lawn technique dos Santos et al. [24] on BHI plates supplemented with 1% agar (*w/v*) containing individually *Salmonella enteritidis* ATCC 13076, *Klebsiella aerogenes* ATCC 13048, *S. aureus* ATCC 29213, *Bacillus cereus* ATCC 11778, *E. coli* ATCC 8739, *Clostridium perfringens* ATCC 13124, *L. monocytogenes* ATCC 7644, *L. monocytogenes* 408 serovar 1/2c, *L. monocytogenes* 103 serovar 1/2a, *L. monocytogenes* 409 serovar 1/2a, *L. monocytogenes* 302 serovar 4b, *L. monocytogenes* 620 serovar 4b, *L. monocytogenes* 211 serovar 4b, *L. monocytogenes* 106 serovar 1/2a, *L. monocytogenes* 506 serovar 1/2a, *L. monocytogenes* 422 serovar 1/2c, *L. monocytogenes* 603 serovar 1/2b, *L. monocytogenes* 101 serovar 4b, *L. monocytogenes* 712 serovar 1/2c, and *L. monocytogenes* 724 serovar 4b (strains from the collection of the Food Microbiology Laboratory of FCF, USP, São Paulo, SP, Brazil) at an approximate concentration of $10^{5-6}$ UFC/mL. The plates were incubated for 24 h at 37°C, and the formation of inhibition zones was observed. Inhibition zones with a minimum of 3 mm were considered to be potential bacteriocin producers.

### 2.2 Identification and Differentiation of Bacteriocinogenic Isolates

The isolates with antimicrobial capacity were differentiated and identified after being subjected to growth in 9 mL of MRS broth (Difco) for 24 h at 37°C. Immediately after, the DNA of each isolate was extracted using the commercial Quick‐DNA Fungal/Bacterial Miniprep Kit (Zymo Research, Irvine, CA, USA), according to the manufacturer's recommendations. The concentration and quality of the DNA obtained were evaluated in a NanoPhotometer (Implen, Westlake Village, CA, USA).

The isolates were also differentiated by repPCR with primer (GTG)_5_ listed to Table 1, Castilho et al. [25], using the Veriti 96 thermocycler (Applied Biosystems, Foster City, CA, USA) as follows: denaturation step at 95°C for 5 min, 95°C for 30 s, annealing at 40°C for 30 s, and extension at 65°C for 8 min in 30 cycles, with a final extension at 65°C for 16 min concluding the amplification. The amplicons were then separated and analyzed using the fingerprint technique in gel electrophoresis separation, 1.5% agarose with SYBR Safe DNA Gel Stain (Thermo Scientific), in a 100 V run for 45 min, and visualized on the Molecular Imager GelDoc XR (Bio‐Rad, Hercules, CA, USA).

**TABLE 1:** Primers targeting different genes associated with beneficial and virulence properties of *S. epidermidis* ST0409KOC used in the current analyses, amplicon sizes, and obtained results (+ : gene was detected in DNA from *S. epidermidis*; ‐ : no evidences for presence of the gene in DNA from *S. epidermidis*).

Gene	Primer	Sequence	Size	*S. epidermidis*	References
***Genes related to differentiation and identification***					
	(GTG)_5_	GTGGTGGTGGTGGTG		Differentiation	[25]
***16S***	BSF8/20‐ F	AGAGTTTGATCCTGGCTCAG	1518	Identification	[26]
	BSR 1541/20‐R	AGGAGGTGATCCAGCCGCA			
***Bacteriocin‐related genes***					
***entA***	entA‐F	GAGATTTATCTCCATAATCT	452	−	[27]
	entA‐R	GTACCACTCATAGTGGAA			
***entB***	entB‐F	GAAAATGATCACAGAATGCCTA	159	−	
	entB‐R	GTTGCATTTAGAGTATACATTTG			
***entL50B***	entL50B‐F	ATGAGAAAAAAATTATTTAGTTT	216	−	
	entL50B‐R	TTAATGTCCCATACCTGCCAAACC			
***entP***	entP‐F	ATGAGAAAAAAATTATTTAGTTT	216	−	
	entP‐R	TTAATGTCCCATACCTGCCAAACC			
***pedPA‐1***	PedPro‐F	CAAGATCGTTAACCAGTTT	1238	−	[32]
	Ped1041‐R	CCGTTGTTCCCATAGTCTAA			
***plc A***	PlcA‐F	CTGCTTGAGCGTTCATGTCTCATCCCC	245	−	[38]
	PlcA‐R	ATGGGTTTCACTCTCCTTCTAC			
***lgnA***	lgnA‐F	ATTTAATACGGACGGTATTGAT	205	−	[33]
	lgnA‐R	GGAGT AAAAAGATGGAAAACAA			
***lgal***	lgaI‐F	AGAAAATGGGCTAACTCCGG	261	−	
	lgaI‐R	ATGAATAAAACAGAAATAATGACT			
***gak***	gak‐F	CGTAATTCGAGCTCCACCTCTGCT GTT TTTC	341	−	[34]
	gak‐R	AGACTTTGCAAGCTTGCAATATTACGTTTGTGGG			
***gak(R1)***	gak‐F	CGTAATTCGAGCTCCACCTCTGCTGTT TTTC	350	−	
	gak‐R1	AGACTTTGCAAGCTTTTAATCCTGACTCATCAGATATTC			
***lcn972***	Lcn972‐F	TTGTAGCTCCTGCAGAAGGAACATGG	232	−	[35]
	Lcn972‐F	GCCTTAGCTTTGAATTCTTACCAAAAG			
***lcn‐gq***	LcnGQ‐F	GAAAGAATTATCAGAAAAAG	382	−	
	LcnGQ‐R	CCACTTATCTTTATTTCCCTCT			
***epiNI01***	epi NI01/F	ATATAT TACATATGGCAGCATTTATGAAGTTAATTCAG	152	**+**	[36]
	epi NI01/R	TACGTTCTCGAGTTATTATGCCCATAATTTTTTGATTTG			
***aurA70***	aur A70/F	GATTAAACCTTATAATAGA	520	−	[37]
	aur A70/R	CTAATAATAAAATATTAACAA			
***aurA53***	aur A53/F	GAAGTTATGAAAACTATA	490	−	
	aur A53/R	CATAAAACAAAGAACCAAAGT			
***pRJ6***	pRJ6/F	GAGTGGAGAAACAGTAGT	855	−	
	pRJ6/R	CTATTCTTCCCAATTCAT			
***Vancomicin‐related genes***					
***van*A**	VanA‐F	GTAGGCTGCGATATTCAAAGC	231	−	[31]
	VanA‐R	CGATTCAATTGCGTAGTCCAA			
***van*B**	VanB‐F	GTAGGCTGCGATATTCAAAGC	330	−	
	VanB‐R	GCCGACAATCAAATCATCCTC			
***van*C**	VanC‐F	ATCCAAGCTATTGACCCGCT	402	−	
	VanC‐R	TGTGGCAGGATCGTTTTCAT			
***van*D**	VanD‐F	TGTGGGATGCGATATTCAA	500	−	
	VanD‐R	TGCAGCCAAGTATCCGGTAA			
***van*E**	VanE‐F	TGTGGTATCGGAGCTGCAG	513	−	
	VanE‐R	GTCGATTCTCGCTAATCC			
***van*G**	VanG‐F	GAAGATGGTACTTTGCAGGGCA	519	−	
	VanG‐R	AGCCGCTTCTTGTATCCGTTTT			
***Virulence‐related genes***					
***IS*16**	IS16‐F	CATGTTCCACGAACCAGAG	547	−	[39]
	IS16‐R	TCAAAAAGTGGGCTTGGC			
***ace***	Ace‐F	GAATTGAGCAAAAGTTCAATCG	1008	−	[40]
	Ace‐R	GTCTGTCTTTTCACTTGTTTC			
***efa***	Efa‐F	GCCAATTGGGACAGACCCTC	688	−	
	Efa‐R	CGCCTTCTGTTCCTTCTTTGGC			
***esp***	Esp‐F	AGATTTCATCTTTGATTCTTGG	510	−	[41]
	Esp‐R	AATTGATTCTTTAGCATCTGG			
***asa***	Asa‐F	GCACGCTATTACGAACTATGA	375	−	
	Asa‐R	TAAGAAAGAACATCACCACGA			
***hyl***	hyl‐F	ACAGAAGAGCTGCAGGAAATG	276	−	
	hyl‐R	GACTGACGTCCAAGTTTCCAA			
***hdc***	hdc‐F	AGATGGTATTGTTTCTTATG	367	−	[42]
	hdc‐R	AGACCATACACCATAACCTT			
***tdc***	tdc‐F	GAYATNATNGGNATNGGNYTNGAYCARG	924	−	
	tdc‐R	CCRTARTCNGGNATAGCRAARTCNGTRTG			
***odc***	odc‐F	GTNTTYAAYGCNGAYAARCANTAYTTYGT	1446	−	
	odc‐R	ATNGARTTNAGTTCRCAYTTYTCNGG			
***sed***	sed/F	TTCGAAATGCTGATGGTTGT	1113	−	[43]
	sed/R	AGCTATCATCAATTTCTTTTCAAGC			
***seb***	seb/F	TGATGATAATCATGTATCAGCA	850	−	
	seb/R	ACGGCGACACAGTAACTATCCA			
***sec***	sec/F	TCAAGATGCTTAGAAATCCTCTGT	1095	−	
	sec/R	TCGGTGCTTGCCTTTTTAGGA			
***IS*256**	IS256/F	TGAAAAGCGAAGAGATTCAAAGC	1101	−	[44]
	IS256/R	ATGTAGGTCCATAAGAACGGC			
***IS*257**	IS257/F	GCTAATTTCGTGGCATGGCG	620	**+**	
	IS257/R	GTTATCACTGTAGCCGTTGG			
***IS*1272**	IS1272/F	GCTCGTTGAGCTACTTTTC	1820	−	
	IS1272/R	CCTAGAGAAATAGCCAGTAAATG			
***sesl***	SesI F1	GTAGGATCCTATGTAAATGACTCAAATGTT	501	−	[45]
	SesI R1	TGAGTCGACTTATTTAAGAATTAGAAGTACG			
***cylA***	cylA‐F	ACTCGGGGATTGATAGGC	688	−	[41]
	cylA‐R	GCTGCTAAAGCTGCGCTT			
***gel***	Gel‐F	TATGACAATGCTTTTTGGGAT	213	−	
	Gel‐R	AGATGCACCCGAAATAATATA			

For identification, primers BSF8‐20 F and BSR1541‐20 R (Table 1) were used to target conserved ribosomal regions (16s), as recommended by Héquet et al. [26]. PCR reactions were performed in a Veriti 96 thermocycler (Applied Biosystems) under the following conditions: denaturation step at 94°C for 5 min, followed by 35 cycles at 94°C for 10 s, annealing at 61°C for 20s, extension at 68°C for 2 min, and final extension at 72°C for 7 min. The generated amplicons were separated by electrophoresis in 1% (*w/v*) agarose gel supplemented with SYBR Safe DNA Gel Stain (Thermo Scientific) under running conditions of 100 V for 45 min. The images were visualized on a Molecular Imager GelDoc XR (Bio‐Rad) apparatus. Subsequently, the amplicons were sequenced in an outsourced service at the Institute of Biomedical Sciences, University of São Paulo, Center for Human Genome and Stem Cell Studies using the Sanger DNA sequencing technique (BigDye Terminator v3.1 Cycle Sequencing Kit) and identified by Sequencing Analysis 7.0 (Base Caller KB). The received sequences were then analyzed in the Basic Local Alignment Search Tool (BLAST, National Center for Biotechnology Information, Bethesda, MD, USA).

### 2.3 Safety Assessment of Bacteriocinogenic Isolates

Based on the differentiation and identification set of different bacterial cultures was selected for further evaluation. However, in the current study, only results for the non‐LAB were explored and presented. Further data on the identified isolates remain confidential. For all safety tests, a non‐LAB selected in the current study, a *S. epidermidis* ST0409KOC, was cultured in MRS broth (Oxoid) for 24 h at 37°C, except for the production of biogenic amines, a specific condition described below.

### 2.4 Hemolytic Activity

*S. epidermidis* ST0409KOC was activated in 9 mL of MRS broth (Difco) for 24 h at 37°C and subjected to hemolytic activity testing according to the recommendations of Fugaban et al. [27]. Evaluated strain was plated on Sheep Blood Agar (Laborclin, Sao Paulo, SP, Brazil), and incubated for 24 h at 37°C. Halo formation was observed and classified as γ‐hemolytic (absence of halo), α‐hemolytic (green halos), and β‐hemolytic (translucent halos). *L. monocytogenes* ATCC 7644 and *Lactiplantibacillus plantarum* ST8Sh were used as a positive control for α‐hemolytic and γ‐hemolytic activity, respectively.

### 2.5 Mucin Degradation

The mucin degradation test was performed according to Rwubuzizi et al. [21]. The MRS culture medium was prepared manually according to the commercial composition, but with and without glucose supplemented with 1.5% agar (*w/v*), 0.3% MGP (*w/v*) (pig gastric mucin, type III, Sigma‐Aldrich, Spruce Street, Saint Louis, MO, USA), and without and with 1% glucose (*w/v*). Both media were autoclaved for 15 min at 121°C and distributed in sterile plates. Ten microliters of the investigated strain, *S. epidermidis* ST0409KOC, were inoculated onto the surfaces and incubated for 72 h at 37°C. The plates were then stained for 30 min with 0.1% amino black (Sigma Aldrich) (*m/v*) in 3.5 M acetic acid (Synth, Diadema, SP, Brazil). Finally, the plates were washed with 1.2 M acetic acid, and the lytic zones around the colonies were evaluated for degradation capacity. *B. cereus* ATCC 11778 was used as a positive control.

### 2.6 Resistance/Acquiescence to Antibiotics

In the evaluation of the resistance/acquiescence to antibiotics, metabolite active culture of *S. epidermidis* ST0409KOC was evaluated following the recommendations from EFSA [28] with minimal inclusions, according to the recommendations from Rwubuzizi et al. [21]. Evaluated strain, *S. epidermidis* ST0409KOC, was seeded on MRS agar (Oxoid) to a final concentration of 10^6–7^ CFU/mL. Sequentially, antibiotic disks impregnated with following antibiotics vancomycin (30 µg/disc), streptomycin (10 µg/disc), gentamicin (10 µg/disc), ofloxacin (5 µg/disc), ampicillin (20 µg/disc), chloramphenicol (30 µg/disc), erythromycin (15 µg/disc), tylosin (60 µg/disc), amoxicillin (10 µg/disc), tetracycline (30 µg/disc), and kanamycin (30 µg/disc), all from the Cefar (Cefar Diagnóstica Ltda, Sao Paulo, SP, Brazil) were placed on the surface and plates incubated for 24 h at 37°C. Plates were observed and results recorded as the diameter of the recorded inhibition zones.

### 2.7 Production of Biogenic Amines

Following the recommendations described by Bover‐Cid and Holzapfel [29], *S. epidermidis* ST0409KOC was cultured in MRS broth supplemented with precursors for biogenic amine production in proportions of 1:100 (tyrosine, ornithine, lysine, and histidine, all from Sigma‐Aldrich) and 0.005% pyridoxal‐5‐phosphate (*w/v*) (Sigma‐Aldrich) for 24 h at 37°C for five subsequent times. MRS plate (Oxoid) was supplemented with 2% agar (w/v), and specific precursors for biogenic amine production were prepared, followed by incubation for up to 7 days at 37°C and observation of possible color changes, evidence for the change of pH, and consequences for the production of biogenic amines. *B. cereus* ATCC 11778, *Lpb. plantarum* ST8Sh, and *L. monocytogenes* ATCC 7644 served as controls.

### 2.8 Antimicrobial Properties

#### 2.8.1 Protein Validation of Antimicrobial Activity

In order to confirm the protein nature of the antimicrobials produced by *S. epidermidis* ST0409KOC, their supernatant was exposed to enzymatic action following the methodology of Valledor et al. [23]. The supernatant was individually subjected to 0.1 mg/mL of Proteinase K (final concentration), incubated for 1 h at 37°C, followed by heat treatment for 3–5 min at 98–100°C to stop the enzymatic reaction. Then, the residual antimicrobial activity (AU/mL) was evaluated against *L. monocytogenes* 211 serovar 4b (L211), *L. monocytogenes* 422 serovar 1/2c (L422), and *L. monocytogenes* 603 serovar 1/2b (L603) under conditions previously described. The supernatant not treated with Proteinase K served as a positive control.

#### 2.8.2 Bacteriocin Stability

To evaluate the stability of the bacteriocin produced by *S. epidermidis* ST0409KOC, its supernatant was exposed to different temperatures, pH, and chemical conditions, according to the methodology of dos Santos et al. [24]. To assess the influence of selected chemicals, the supernatant was exposed to 1% NaCl, SDS, Tween 80, Tween 20, and skim milk for 1 h at 37°C. In temperature variation, the supernatants were incubated at 8°C, 30°C, 37°C, 60°C, and 98°C for 1 h and 121°C for 15 min. Finally, to vary the pH, the supernatant had its pH altered with sterile 1 M NaOH or 1 M HCl to levels of 2.0, 4.0, 6.0, 8.0, or 10.0. Samples were incubated for 1 h at 37°C, and the pH was readjusted to 5.0–7.0 with 1 M NaOH or 1 M HCl, as needed. All experimental variations were then tested for residual bacteriocin activity against *L. monocytogenes* 211, *L. monocytogenes* 422, and *L. monocytogenes* 603, following the same incubation method described previously, and AU/mL were determined as previously described by Valledor et al. [23]. Untreated supernatant was used as a positive inhibition control.

#### 2.8.3 Growth, Acidification, and Bacteriocinogenic Activity Kinetics

Growth kinetics, acidification, and bacteriocin production of *S. epidermidis* ST0409KOC were performed following the methodology described by Valledor et al. [23]. The *S. epidermidis* ST0409KOC strain was incubated at 5% (*v/v*) levels for 24 h at 37°C in MRS broth (Oxoid) from a culture previously activated for 18 h. The growth of *S. epidermidis* ST0409KOC was evaluated every 1 h based on the change in optical density (OD), measured at 600 nm in an Ultrospec 2000 (Pharmacia Biotech, Marlborough, MA, USA), simultaneously with the recording of the pH change in a pH meter (Láctea Aparelhos Científicos e Eletrônicos LTDA, São Paulo, SP, Brazil). In addition, to evaluate the produced bacteriocin in a monitored period over 24 h, aliquots were collected every 3 h. Cell free supernatant was prepared as described before and serially diluted 2× in 100 mM potassium phosphate buffer (pH 6.5) and the volume of 10 µL spotted on plates containing *L. monocytogenes* 211, *L. monocytogenes* 422, and *L. monocytogenes* 603, according to the method previously discussed. The bacteriocinogenic activity capacity was expressed in AU/mL (Arbitrary Units), following the mathematical formula described by Valledor et al. [23]: type of dilution (***D*** = 2×), first dilution that did not present zone of inhibition (*n*) and inoculated volume (***P*** = 10 µL), represented below.

$$\frac{\mathrm{AU}}{\mathrm{mL}}=\frac{D^{n}\times 1000}{P}$$

#### 2.8.4 Action of the Bacteriocin Produced by *S. epidermidis* ST0409KOC on the Growth of *L. monocytogenes* Strains

First, the cultures of *L. monocytogenes* 211, *L. monocytogenes* 422, and *L. monocytogenes* 603 were activated and cultured in BHI broth (Oxoid) for 24 h at 37°C, in order to obtain fresh cultures for later analysis. Again, they were subjected to growth in 200 mL BHI broth (Oxoid) with 1% inoculum (*v/v*) and incubated at 37°C. The cell‐free supernatant of *S*. *epidermidis* ST0409KOC obtained as described before was filtered through a 0.22 µm filter (Kasvi) and added (as 10%, *v/v*) to the cultures of *L. monocytogenes* 211, *L. monocytogenes* 422, and *L. monocytogenes* 603 after 3 h of incubation. The changes in OD at 600 nm were analyzed every 1 h in the Ultrospec 2000 spectrophotometer (Pharmacia Biotech) together with the change in pH by a pH meter (Láctea Aparelhos Científicos e Eletrônicos LTDA) for 12 h. Cultures of *L. monocytogenes* 211, *L. monocytogenes* 422, and *L. monocytogenes* 603 without the addition of supernatant were used as growth control according to the recommendations from Favaro et al. [30].

### 2.9 Screening for the Presence of Genes Related to Bacteriocin Production, Virulence and Safety Determinants, and Beneficial Properties in *S. epidermidis* ST0409KOC

#### 2.9.1 Bacteriocins Associated Genes

After DNA extraction as previously described, the analysis for the presence of bacteriocin‐coding genes was performed in a Veriti 96 thermocycler, following the recommendations described by Fugaban et al. [31], Todorov et al. [32], Maldonado Barragán et al. [33], Telke et al. [34], Mirkovic et al. [35], Sandifort and Upton [36], Brito et al. [37], and Todorov et al. [38], as described in Table 1. The amplicons were separated by electrophoresis (100 V, 45 min) in agarose gel (1%–2%, w/v) supplemented with SYBR Safe DNA Gel Stain (Thermo Scientific) and visualized in Molecular Imager GelDoc XR (Bio‐Rad). The results were evaluated according to the size of the target amplicons.

#### 2.9.2 Virulence Factors, Vancomycin Resistance, and Biogenic Amines Associated Genes

*S. epidermidis* ST0409KOC DNA was investigated for the presence of virulence genes, vancomycin resistance, and production of biogenic amines genes following the recommendations of Fugaban et al. [27], Werner et al. [39], Martín Platero et al. [40], Vankerckho et al. [41], de Las Rivas et al. [42], Johler [43], Kozitskaya et al. [44], and Bowden et al. [45] as described by each author according to Table 1. PCR reactions were performed in a Veriti 96 thermocycler, and amplicons were separated by electrophoresis (100 V, 45 min) in agarose gel (1%–2%, *w/v*) supplemented with SYBR Safe DNA Gel Stain (Thermo Scientific) and visualized in Molecular Imager GelDoc (Bio‐Rad). The results were evaluated taking into account the size of the target amplicons.

#### 2.9.3 Behavior of the Studied Strains in Simulated Stomach‐Duodenal Passage (SSDP) Models

The *S. epidermidis* ST0409KOC was evaluated for its survival capacity when exposed to the SSDP simulation test according to Arellano‐Ayala et al. [46]. *S. epidermidis* ST0409KOC was cultured in 10 mL of MRS broth at 37°C for 24 h, the cells were collected by centrifugation (4000 ×*g*, 5 min, 20°C), resuspended in 10 mL of sterile saline (0.85%) with pH 2.5 and homogenized for 1 min. The cells obtained were incubated at 37°C for 1 h, simulating passage through the stomach (T1). Then, 4 mL of bile salts (10% bile acid) and 17 mL of synthetic pancreatic juice with pH 6.0 and saline composition: 6.4 g/L NaHCO_3_, 0.239 g/L KCl, and 1.28 g/L NaCl were added to simulate the small intestine, incubating again at 37°C for 2 h (T2). The beginning of incubation, the first hour and the third hour represent respectively the initial stage, the stomach conditions and the duodenum conditions, the collected aliquots were used to estimate as CFU/mL by serial dilutions, placed on MRS plates with agar and incubated at 37°C for 48 h.

## 3 Results and Discussion

### 3.1 Isolation of Colonies With Bacteriocinogenic Potential

With the objective to select a safe strain/s with potential beneficial properties, we have combined two principal goals in our study. Primary, in the prescreening for the bacteriocin production strains, we have isolated microbial cultures with antagonistic properties. Moreover, in the second stage of the experimental procedures, we have focused on proving the safety of the selected microbial culture/s. And only after, the safe and bacteriocin producers were further evaluated with objective potential application as beneficial/probiotic cultures. Three samples of Bulgarian feta cheese were investigated as a source for the isolation of bacteriocinogenic strains with activity versus *L. monocytogenes*. In the initial assessment, 81 isolates were selected according to the presence of inhibitory zones versus *L. monocytogenes* ATCC 7644. Moreover, the total bacterial count for the studied cheese was between 5.60 × 10^5^ and 3.20 × 10^7^ CFU/mL.

Feta cheese, a popular brined curd cheese, has a characteristic salty and tangy flavor and is preserved under immersion with brine containing NaCl, typically ranging between 5% and 10% [47]. Moreover, the presence of NaCl helps not only to preserve the cheese but enhance its flavor and maintain its texture during storage. Like many dairy products, feta cheese can be a carrier of beneficial or spoilage microorganisms. These can include beneficial bacteria like lactobacilli, which are integral to fermentation and flavor development. However, the microbial load may also include spoilage organisms or, less commonly, pathogens if hygiene during production is not maintained adequately. However, feta's high salt content creates an environment that limits the growth of spoilage or pathogenic microbes. Managing microbial load is critical for both food safety and quality. Proper storage conditions, such as refrigeration and maintaining its brine, are vital to preserving feta cheese and preventing spoilage or contamination.

Among the 81 originally selected isolates, only 17 showed bacteriocinogenic activity when subjected to individual tests against *L. monocytogenes* ATCC 7644 (Supporting Material 1). In preliminary selection tests, cultures considered as bacteriocinogenic were evaluated regarding catalase production, Gram staining (as positive with coccus or rod morphology), γ‐hemolytic, and sensitive to vancomycin. With the objective of investigating a non‐LAB bacteriocinogenic strain, isolates selected for further investigation, as presented catalase catalase‐positive and γ‐hemolytic properties were considered for the following experiments.

### 3.2 Differentiation and Identification

All 17 bacterial isolates were differentiated based on repPCR fingerprinting analysis. From them, two isolates were previously identified as catalase‐positive and were receiving principal attention. However, based on generated fingerprints (data not shown), both isolates, ST0409KOC and ST0411KOC, generated an equal repPCR profile, an argument that can be considered as isolation of the same strain in duplicate. It is important to mention that both isolates originated from different cheese samples but were obtained from the same dairy facility.

Both isolates, ST0409KOC and ST0411KOC were preliminary identified according to their phenotypic, biochemical and physiological characteristics according to recommendations from Bergey's Manual de Vos et al. [22] and further identified by 16s rRNA using primers BSF8‐20 and BSR1541‐20 according to Table 1. Two strains were identified as *S. epidermidis* according to the BLAST analysis (97% identity compared to *S. epidermidis* type strains in GenBank). In the following investigation we have focused on *S. epidermidis* ST0409KOC for subsequent analyses. As *S. epidermidis* has the reputation of being a not safe strain, for use it was academic curiosity to evaluate the safety properties first, and as shown to be considered as safe, the investigated strain was further evaluated for his bacteriocinogenic property. Moreover, bacteriocin production can be even considered as virulence factor, if was associated with no safe strains.

*S. epidermidis* is commonly found on human skin and mucous membranes for humans and other animals [3]. Most probably, its presence in feta cheese can be related as a result of contamination during handling or production, originating from the cheese working personnel or from the animal during milk collection. *S. epidermidis* is generally considered a non‐pathogenic species; however, it can form biofilms and exhibit antibiotic resistance, which could pose challenges in food safety if its levels are significant [10]. Moreover, proper hygiene, pasteurization of milk, and brine's antimicrobial properties are considered critical in minimizing its occurrence in feta cheese. Monitoring microbial load ensures both the safety and quality of the product for consumers.

### 3.3 Safety Tests

*S. epidermidis* ST0409KOC was investigated for its safety in phenotypic and genotypic tests. In the presence of 11 antibiotics recommended by EFSA [28], resistance to certain antibiotics was observed. Strain *S. epidermidis* ST0409KOC was sensitive to different antibiotics such as gentamicin, ampicillin, chloramphenicol, tylosin, amoxicillin, and tetracycline, resulting in a range of alternatives for its microbiological control. However, *S. epidermidis* ST0409KOC showed resistance to other antibiotics under the same studied conditions, with streptomycin, ofloxacin, erythromycin, vancomycin, and kanamycin, antibiotics with no effect on its inhibition (Table 2, Supporting Material 2).

**TABLE 2:** Resistance/sensibility of *S. epidermidis* ST0409KOC to different antibiotics, recommended by EFSA.

Antibiotics	Sensibility
Streptomycin	R
Ofloxacin	S
Erythromycin	R
Kanamycin	R
Vancomycin	S
Gentamicin	S
Ampicillin	S
Chloramphenicol	S
Tylosin	S
Amoxicillin	S
Tetracycline	S

Abbreviations: R, resistance; S, susceptible.

Mucin is found throughout the gastro‐intestinal tract (GIT), making up mucus, which in turn has a primary function of protecting the body's epithelial tissues. Therefore, the mucin degradation test is of great importance in analyzing the ability of bacteria to interact negatively with these tissues. *S. epidermidis* ST0409KOC was exposed to two in vitro conditions, one in solid culture media (SCM) produced in the same way, differing only in the presence or absence of glucose, incubated (37°C, 24 h), and analyzed in the same way. When *S. epidermidis* ST0409KOC was grown on MRS agar plates containing mucin without glucose, it did not demonstrate degradation activity (Supporting Material 3). However, when exposed to an environment containing MRS agar containing mucin and glucose, it was possible to observe the formation of degradation halos around the colony. This can be explained by the activation of genes related to mucin degradation, which can be activated in the presence of glucose.

*S. epidermidis* species have a high affinity for mucin on different surfaces, but their interaction is closely related to adhesion, in the formation of biofilms on catheters, prostheses, and other abiotic and biotic surfaces, as well as in the production of mucin (exopolysaccharides) [1, 3, 10, 48]. According to Jacob et al. [49], although the superficial growth of *S. epidermidis* was greater on plates containing mucin, it was possible to observe the absence of its use as a carbon source (degradation), this occurrence being related to the characteristics of the culture medium (hydration and lubrication of mucin) and secretion of soluble phenolic modulins (SPMs), regulated by *quorum sensing*. Therefore, it is possible to verify that *S. epidermidis* does not have enzymatic mucin degradation abilities but uses its presence to disperse on mucosal surfaces. However, genes related to enzyme production can be shared and transferred horizontally, giving them a new virulence capacity, requiring continuous investigation of new strains to be isolated and identified.

Hemolytic activity among bacteria of the *Staphylococcus* genus is commonly observed as positive, configuring an even more pathogenic profile in bacteria belonging to this genus. Data presented by Xu et al. [50] demonstrate this high prevalence, with *S. aureus*, *S. pasteuri*, *S. haemolyticus*, and *S. warneri* species presenting degradation zones in the hemolysis test. A study conducted by Michelim et al. [51] analyzed the safety of *S. epidermidis* species isolated from clinical samples and volunteers, finding that 35.5% of the isolates presented hemolytic activity.

In contrast, a strain of *S. epidermidis* isolated from *Daqu* by Xu et al. [50] did not demonstrate hemolytic activity (γ‐hemolytic). Similarly, *S. epidermidis* ST0409KOC was shown to have no hemolytic activity when exposed to culture medium with blood, being classified as γ‐hemolytic (Supporting Material 4).

Additionally, *S. epidermidis* ST0409KOC did not produce biogenic amines when exposed for five days to precursors related to their production (tyrosine, ornithine, lysine, and histidine). These, in turn, are related to poisoning when ingested in high quantities or by people with low metabolizing capacity and are commonly produced by bacteria present in meat foods [52]. Coton et al. [53] and Bermudez et al. [54] demonstrate the ability of *S. epidermidis* species to produce biogenic amines is prevalent, with the inability of *S. epidermidis* ST0409KOC to produce them being a positive safety factor. Thus, the results of hemolytic activity and biogenic amines obtained so far confirm the safety of *S. epidermidis* ST0409KOC.

### 3.4 Antimicrobial Properties

#### 3.4.1 Protein Validation and Stability of the Bacteriocin

Analysis of the protein nature of the antimicrobial activity of bacteriocin produced by *S. epidermidis* ST0409KOC was investigated. Cell‐free supernatant obtained from *S. epidermidis* ST0409KOC was obtained as described before and evaluated for the confirmation of its proteinaceous nature and stability under different chemical and environmental conditions.

The antimicrobial capacity of bacteriocin produced by *S. epidermidis* ST0409KOC was abolished in the presence of Proteinase K (0.1 mL/mL). However, the supernatant without the addition of the enzyme (positive control) remained with antimicrobial activity against pathogens, confirming the protein nature of the antimicrobial metabolite produced by *S. epidermidis* ST0409KOC. This is a simple, however, effective approach for confirmation of the periodic nature of antimicrobial metabolites, an approach applied in other research projects investigating bacteriocin production [24, 55].

The effect of different temperatures, pH, and chemicals was also tested for their effect on the stability of bacteriocin produced by *S. epidermidis* ST0409KOC. Bacteriocin produced by *S. epidermidis* ST0409KOC remained stable after being exposed for 1 h at 37°C to pH 2.0, 4.0, 6.0, 8.0, or 10.0, as well as in the presence of 1% NaCl, SDS, Tween 80, Tween 20, and skim milk, and test performed with *L. monocytogenes* 211 serovar 4b, 422 serovar 1/2c, and 603 serovar 1/2c as test microorganism. Similar data were observed in studies carried out by Lima et al. [55], where the stability of bacteriocins produced by *Lactococcus garvieae* and *Enterococcus faecium* species maintained action against different serovars of *L. monocytogenes*. Additionally, the bacteriocin produced by *S. epidermidis* ST0409KOC was tolerant when incubated at temperatures of 8°C, 30°C, 37°C, 60°C, and 98°C for 1 h, and 120°C for 15 min. Because class I and II bacteriocins are small structures of approximately 10 kDa, they are very stable under the influence of high temperatures [24]. These results make bacteriocins a biocontrol means of great interest for the food industry, as they can be used in a wide range of conditions [19, 24, 55, 56].

Furthermore, bacteriocins have a specific target cell action, normally acting against other bacteria phylogenetically close to the producing species [36] and have low toxicity [57] and can be applied in the formulation of food products because they do not interact with the starter cultures and, also, serve as control against different pathogens that cause DTA's, such as *L. monocytogenes* [7, 58]. Data obtained by Sandiford and Upton [36] demonstrated that the bacteriocin produced by a strain of *S. epidermidis* (epidermycin) presented a broad spectrum of inhibition against different strains of the genus *Staphylococcus*, such as methicillin‐resistant *S. aureus* (MRSA), *S. saprophyticus*, *S. hominis*, *S. warneri*, and also against vancomycin‐resistant *Enterococcus* (VRE) *E. faecalis*. These data obtained by different authors reinforce the bacteriocinogenic activity of *S. epidermidis*.

Bacteriocin produced by *S. epidermidis* ST0409KOC was tested against several pathogens, as shown in Table 3. *L. monocytogenes* was the only species affected, affecting different types of serovar, demonstrating the anti‐listeria property of ST0409KOC. Similar data were obtained by Lynch et al. [59], where the bacteriocin capidermycin, produced by *Staphylococcus capitis*, showed action against different serovars of *L. monocytogenes*. Moreover, the activity of bacteriocin produced by *S. epidermidis* ST0409KOC was evaluated versus defend strains of LAB, several of them evaluated as beneficial bacteriocinogenic or putative probiotics strains, from the collection of ProBacLab, Faculty of Pharmaceutical Science, University of São Paulo, São Paulo, Brazil (data not shown). Thus, it is an important point, since industrial application of bacteriocins needs to result in inhibition of pathogens or spoilage microorganisms but will not affect starters and other beneficial cultures.

**TABLE 3:** Antimicrobial effect by bacteriocin produced by *S. epidermidis* ST0409KOC against different pathogens.

Strain	Sensibility
*Salmonella enteritidis* ATCC 13076	−
*Klebsiella aerogenes* ATCC 13048	−
*Staphylococcus aureus* ATCC 29213	−
*Bacillus* cereus ATCC 11778	−
*Escherichia coli* ATCC 8739	−
*Clostridium perfringens* ATCC 13124	−
*L*. *monocytogenes* ATCC 7644	+
*L*. *monocytogenes* 408 serovar 1/2c	−
*L*. *monocytogenes* 103 serovar 1/2a	−
*L*. *monocytogenes* 409 serovar 1/2a	−
*L*. *monocytogenes* 302 serovar 4b	−
*L*. *monocytogenes* 620 serovar 4b	−
*L*. *monocytogenes* 211 serovar 4b	+
*L*. *monocytogenes* 106 serovar 1/2a	−
*L*. *monocytogenes* 506 serovar 1/2a	−
*L*. *monocytogenes* 422 serovar 1/2c	+
*L*. *monocytogenes* 603 serovar 1/2b	+
*L*. *monocytogenes* 101 serovar 4b	−
*L*. *monocytogenes* 712 serovar 1/2c	−
*L. monocytogenes* 724 serovar 4b	−

#### 3.4.2 Growth Kinetics and Bacteriocinogenic Activity

**FIGURE 1:**
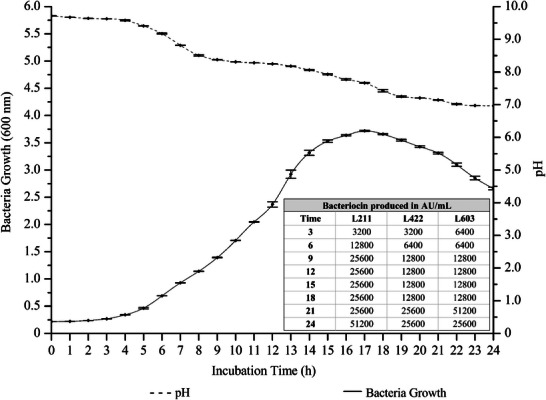
Dynamic of bacterial growth of *S. epidermidis* ST0409KOC, cultured in MRS at 37°C (solid line) (evaluated as changes in OD at 600 nm), acidification (dotted line) (evaluated as changes in pH), and bacteriocin production (bars) (recorded as AU/mL) evaluated versus *L. monocytogenes* 211, 422 and 603.

Growth of *S. epidermidis* ST0409KOC was monitored when cultured in MRS broth at 37°C for 24 h (Figure 1). In addition, acidification and bacteriocin production were recorded for the same monitored period. The growth of *S. epidermidis* ST0409KOC was observed visually and recorded spectrophotometrically due to the increasing turbidity of the g, in which it presented a higher peak of cell density after 17 h of growth (OD of 3.746, value obtained after recalculation taking in consideration applied dilution) (Figure 1). As shown in Figure 1, bacteriocin produced by *S. epidermidis* ST0409KOC presented maximum values of 51 200 AU/mL in 21 h and 24 h of growth, recorded against *L. monocytogenes* 603 and *L. monocytogenes* 211, respectively. Furthermore, bacteriocin produced by *S. epidermidis* ST0409KOC showed values of 25 600 AU/mL against *L. monocytogenes* 422 in same time points of the cultivation. These results indicate that the bacteriocinogenic activity of *S. epidermidis* ST0409KOC is benefited by the longer incubation period, that is, a greater antimicrobial profile is present in the logarithmic decreasing phase.

Our observations agree with previous reports by dos Santos et al. [24], Lima et al. [55], as bacteriocin production is often closely associated with the stationary phase of bacterial growth, where nutrients in the environment are depleted, and bacteria enter a state of metabolic stress, which may trigger antimicrobial metabolite production. The transition to the stationary phase often induces gene expression responsible for bacteriocin synthesis, ensuring efficient use of energy and resources in challenging conditions [60].

Producing bacteriocins in the stationary phase may offer some benefits for the producing cells, including allowing bacteria to conserve energy by focusing on survival rather than rapid reproduction, making the production of bacteriocins a strategic adaptation. Bacteriocins can inhibit competing microorganisms at a point where microbial competition may be more intense, thus enhancing their effectiveness in microbial defense. Moreover, in food biotechnology, bacteriocins are of great interest for their ability to naturally preserve food products by inhibiting spoilage and pathogenic bacteria, offering an eco‐friendly alternative to synthetic preservatives. Their production kinetics and mode of action make them particularly valuable in food safety and quality control [56].

#### 3.4.3 Action of Bacteriocin Produced by *S. epidermidis* ST0409KOC on the Growth of *L. monocytogenes*

*L. monocytogenes* is a Gram‐positive, non‐spore‐forming, catalase‐positive, and β‐hemolytic pathogen in the presence of blood, and is found in various environments such as water, vegetation, soil, sewage, and human feces [61]. *L. monocytogenes* is of great concern in the food industry due to its broad spectrum of survival conditions and its high virulence rate [59]. This is possible due to its ability to form biofilms and adhere to the surfaces of food‐producing plants and other abiotic surfaces, as well as to adhere to food [62]. In addition, *L. monocytogenes* survives a wide range of temperatures (−0.4°C to 45°C), pH (4.6 to 9.5), low water activity (aW <0.90), and salt concentrations up to 20% [61]. Pregnant women are the main target audience due to the abortive capacity of *L. monocytogenes*, accompanied by immunocompromised individuals such as newborns, transplant recipients, and the elderly [55].

**FIGURE 2:**
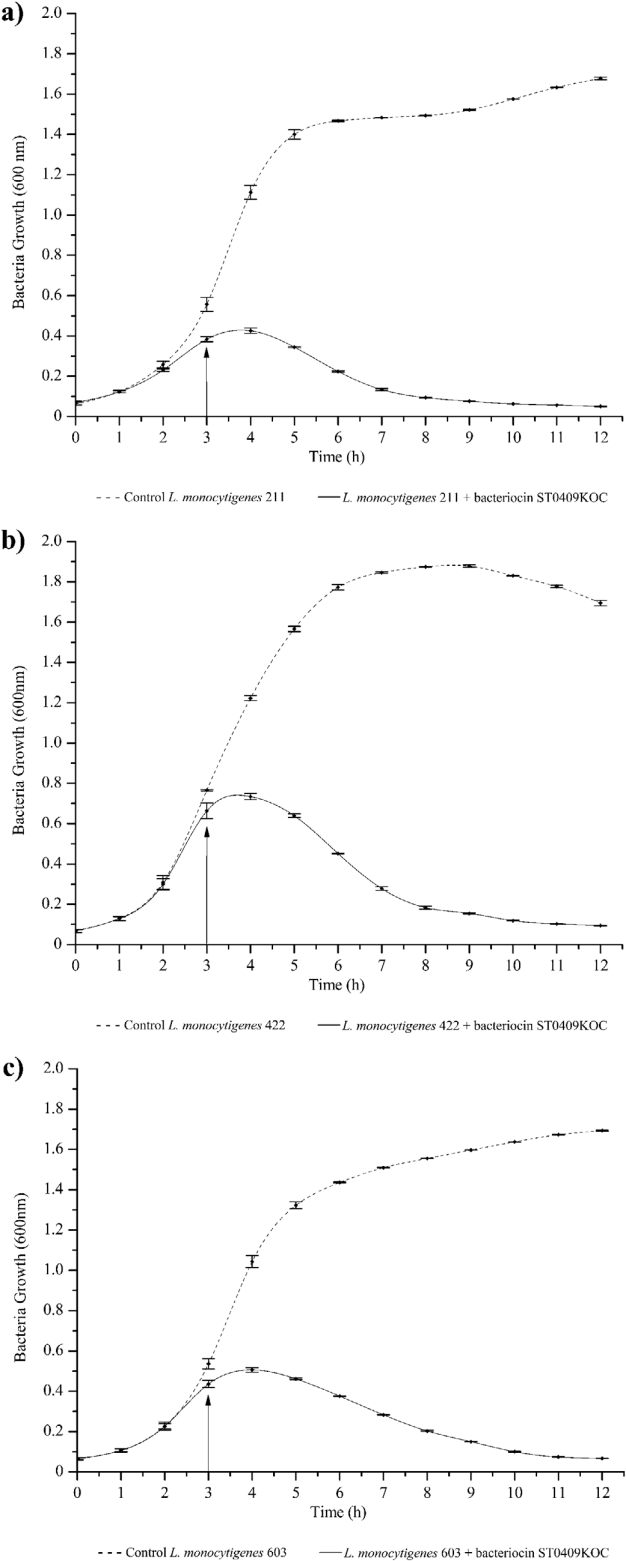
Bacterial growth inhibition of *L. monocytogenes* 211, 422, and 603 by bacteriocin produced by *S. epidermidis* ST0409KOC. The arrow represents when bacteriocin ST0409KOC was added at 3 h of incubation, except for the controls.

Strains of *L. monocytogenes* 211 serovar 4b, *L. monocytogenes* 422 serovar 1/2c, and *L. monocytogenes* 603 serovar 1/2b were exposed in their logarithmic phase to bacteriocin produced by *S. epidermidis* ST0409KOC in broth, observing their growth for 12 h (Figure 2). *S. epidermidis* ST0409KOC showed quantitatively distinct bacteriocinogenic activity against the *L. monocytogenes* tested, with the highest activity (51 200 AU/mL) against *L. monocytogenes* 211 and 603 at 24 and 21 h, respectively. Bacteriocinogenic activity against *L. monocytogenes* 603 was the only one that showed a decrease in test organisms at the end of its growth. ST0409KOC bacteriocin inhibited the growth of the three *L. monocytogenes* analyzed after inoculation (Hour 3), during the following 9 h of analysis, as shown in Figure 2. Nevertheless, it is possible to verify that the bacteriocin produced by *S. epidermidis* ST0409KOC was able to inhibit the growth of the *L. monocytogenes* analyzed at levels similar to or lower than the initial ones in question. Similar data were obtained by Lima et al. [55], by inhibiting the growth of *L. monocytogenes* for a period of 12 h, with strains of *Lc. garvieae* and *E. faecium* isolated from the production environments of a Brazilian cheese factory. The origin of these bacteriocinogenic strains can be better explored, since many studies end up investigating food matrices individually, but it is known that strains with beneficial properties can be found in abiotic food production sites [7].

Bacteriocins generally act against microorganisms phylogenetically close to the producing bacteria, in which their production goes beyond bacteria considered safe [63, 64]. A strain of *S. aureus* may present bacteriocinogenic properties by producing different staphylococci, more specifically aureucin, and inhibiting a pool of bacteria, as demonstrated by Navaratna et al. [65], where they identified bacteriocinogenic activity of their strain of *S. aureus* against 120 strains of its same species and numerous other multidrug‐resistant antibiotic bacteria. However, this inhibitory property can be considered a virulence factor in opportunistic and/or pathogenic bacteria because it allows their propagation in different environments by preventing the growth of other microorganisms [24].

*S. epidermidis*, as already mentioned, is a natural culture and a widely colonizer of human epithelial tissue, and under certain conditions may present undesirable clinical effects in compromised humans due to its opportunistic capacity. *S. epidermidis*, in a way, when present in nosocomial infections, can better establish itself in catheters due to its ability to form biofilm, a virulence factor. The ability to synthesize bacteriocins gives this strain an advantage in establishing itself in different regions, being able to prevent the presence not only of pathogenic microorganisms, but also of other previously common residents. This ability may be one of the reasons for its widespread colonization on human skin. Furthermore, since *S. epidermidis* has bacteriocinogenic properties, its presence in food may cause concern to the food industry, since it may have the ability to inhibit the growth of starter cultures, which are responsible for developing the product, giving it specific characteristics such as flavor, texture, aroma, and shelf life. Therefore, bacteriocins often have excellent applications as biocontrol, but with the horizontal sharing of different genes and related genes between different bacteria, certain strains may become more virulent.

### 3.5 Beneficial and Virulence Genes Present in *S. epidermidis* ST0409KOC

#### 3.5.1 Bacteriocin‐Associated Genes

DNA extracted from *S. epidermidis* ST0409KOC was examined for the presence of genes related to bacteriocin production using the primers already mentioned in the literature (Table 1). *S. epidermidis* ST0409KOC gene was further investigated for the presence of other bacteriocin‐related genes commonly found in bacteria of the *Staphylococcus* genus and other bacteriocin genes conform described in Table 1, such as epicidin 280 [66], epidermycin NI01 [36], epilancin K7 [67], pep5 [68], and epilancin 15X [69], in which *S. epidermidis* ST0409KOC generated positive evidence for the presence of epidermycin NI01 gene (Supporting Material 5), which may be responsible for its bacteriocinogenic activity.

The bacteriocin produced by *S. epidermidis* ST0409KOC, as mentioned above, was stable when exposed to different conditions of temperature, pH, and some chemicals. Broad stability data were obtained by Sandiford and Upton [36], where they investigated the stability of the bacteriocin epidermycin NI01 in the presence of lysozyme, lipase, and α‐amylase and observed no enzymatic influence, as well as in the presence of a broad concentration of pH 2.0–10.0 and temperature at 80°C for 60 min, there was no decrease in the activity of epidermycin NI01.

#### 3.5.2 Virulence Genes

DNA extracted from *S. epidermidis* ST0409KOC was analyzed for the presence of different genes related to virulence and vancomycin resistance. Strains of the *S. epidermidis* species often do not present a variety of virulence genes, in many cases being limited to genes related to biofilm formation [10]. *S. epidermidis* ST0409KOC did not present any of the commonly researched virulence genes, namely: vancomycin resistance (*van*A, *van*B, *van*C, *van*D, *van*E, and *van*G); IS*16* (Enterococcal pathogenicity islands); *cyl* (cytolysin); *ace* (collagen protein adhesin); *efa* (endocarditis‐specific protein); *esp* (enterococcal surface protein); *asa* (aggregation protein); *hyl* (hyaluronidase); *hdc*, *odc*, and *tdc* (biogenic amines); and *gel* (gelatinase) according to Table 1.

Furthermore, *S. epidermidis* ST0409KOC did not show resistance to vancomycin when exposed to the test with antibiotic discs. The inability to produce biogenic amines was confirmed by the absence of the *hdc*, *odc*, and *tdc* genes, as well as the non‐production of biogenic amines when exposing *S. epidermidis* ST0409KOC to a culture medium with precursors for biogenic amine production for 5 days. Phenotypic tests are necessary to confirm different properties present in bacteria, since genes present may be silenced, or the production of different substances occurs by other unconventional pathways.

However, the intense interaction of CoNS present in horizontal gene transfer makes these mostly safe strains, such as *S. epidermidis*, acquire virulence abilities, and can be used in different environmental conditions [4]. Strains of *S. epidermidis* have a high affinity for interaction with *S. aureus* species, the latter known to have the ability to produce different toxins, as well as transfer pathogenicity islands to other strains, in addition to being able to have resistance to methicillin [70]. A factor described by Servern and Horswill [4], MRSA can transfer these factors to other strains of *S. epidermidis*, giving rise to methicillin‐resistant *S. epidermidis* (MRSE), where these situations are supported by the genetic flexibility present in the *S. epidermidis* species.

Therefore, *S. epidermidis* ST0409KOC was investigated for virulence genes prevalent in the species, which are: *sed* (staphylococcal enterotoxin D), *seb* (staphylococcal enterotoxin B), *sec* (staphylococcal enterotoxin C), IS*256* (insertion sequence 256), IS*257* (insertion sequence 257), IS*1272* (insertion sequence 1272), *sesl* (*S. epidermidis* surface protein I) according to data presented in Table 1.

Insertion sequences can control some genetic processes and mechanisms, depending on their location, playing a role in the genetic mobility of the microorganism, being able to express a previously silenced gene, or even transpose a genetic selection in other regions [44]. Among the virulence genes commonly found in the bacteria of the *Staphylococcus* genus studied, IS*257* was the only one present in the DNA of *S. epidermidis* ST0409KOC, which is widely found in species of *S. epidermidis*. The IS*257* gene is associated with the dissemination of Tn4003 transposin‐like elements, found in plasmids of *S. aureus* and in CoNS. In addition, IS*257* promotes the transcription of the *thy*E‐*dfr*A‐*orf*‐140 operon, in which the *dfr*A gene present is responsible for encoding the enzyme dihydrofolate reductase (DHFR), producing an altered version resistant to the antibiotic trimethoprim [71]. Therefore, IS*257* is strongly related to bacterial resistance to the antibiotic trimethoprim, preventing its action in blocking bacterial DNA and RNA synthesis [4, 72].

According to a study conducted by Kozitskaya et al. [4], when isolating 230 strains of *S. epidermidis* from different sources, 139 of which were commensal (nasal swabs from volunteers) and 91 clinical (53 from blood cultures and 38 from urinary tract infections), the presence of IS*257* was observed in 204 of the 230 isolates, confirming its widespread presence in this species from different environments. Gene expression should always be investigated, since the presence of a gene does not necessarily configure its expression, since it may be in silenced regions. Therefore, IS*257* identified in the DNA of *S. epidermidis* ST0409KOC should be further studied regarding its expression.

Nowadays, there are studies showing the efficacy of *S. epidermidis* strains with probiotic potential on human skin. According to Wang et al. [73], it was possible to observe the action of *S. epidermidis* against *Propionibacterium acnes*, the latter microorganism related to acne lesions. The inhibition process occurs through the fermentation of glycerol by *S. epidermidis* and consequent production of short‐chain fatty acids (SCFAs). Furthermore, Mottin and Suyenaga [74] show that specific strains of *S. epidermidis* demonstrated beneficial properties on the skin against *S. aureus*, inhibiting its biofilm formation, stabilizing existing cells, as well as stimulating skin defense responses against the pathogen.

The action of *S. epidermidis* is not limited to only against other microorganisms. A study conducted by Balasubramaniam et al. [75] observed that when administering a strain of *S. epidermidis*, it demonstrated beneficial effects against damage caused to the skin by UV‐B, when fermenting LCC (Liquid coco‐caprylate/caprate, a compound present in different cosmetics), producing SCFAs and electricity, reducing the damage caused by UV‐B in the skin of mice. Negari et al. [76] show the property of *S. epidermidis* in promoting the synthesis of type I collagen when fermenting a cosmetic compound, cetearyl isononanoate (CIN), producing SCFAs (butyrate in greater quantity), and inducing collagen synthesis in vitro and in vivo.

Therefore, *S. epidermidis* ST0409KOC can be considered a safe strain because it presents only one virulence gene, which still needs to be further investigated regarding its expression in bioinformatics analyses. Thus, *S. epidermidis* ST0409KOC is a strong candidate for use as a probiotic in different applications, and further studies are needed.

## 4 Conclusion

The production of bacteriocins by *S. epidermidis* ST0409KOC represents a remarkable alignment of nature's ingenuity and human‐driven fermentation processes. Stripped of virulence determinants, this strain sheds its identity as a pathogen and emerges as a potential ally, offering its antimicrobial prowess for the greater good. The ability to produce bacteriocins—a natural defense against competing microorganisms—ushers in a new perspective, where even former adversaries of human health can find purpose in contributing to food safety and pharmaceutical innovation. Crossing points between safety and beneficial properties highlights the transformative power of context and application, where the current *S. epidermidis* ST0409KOC strain is an example where microorganisms can be repurposed from threats to protectors. For the food industry, bacteriocins from *S. epidermidis* ST0409KOC could play a role in preserving products, reducing reliance on synthetic additives, and supporting cleaner production practices. Moreover, in pharmaceuticals, these bacteriocins could inspire novel antimicrobial therapies, aiding humanity's fight against resistant pathogens and improving skin health.

## Funding

This project was supported by the São Paulo Research Foundation (FAPESP) (grants 2023/05394‐9, 2024/14944‐2); by the Centre for Research and Development in Agrifood Systems and Sustainability, funded by FCT (UIDB/05937/2020 and UIDP/05937/2020), Fundação para a Ciência e a Tecnologia, Portugal.

## Ethics Statement

This article does not contain any studies on human or animal subjects.

## Conflicts of Interest

The authors declare no conflicts of interest.

## Supporting information




**Supplementary File**: mnfr70359‐sup‐0001‐SuppMat.zip.

## Data Availability

The data that support the findings of this study are available from the corresponding author upon reasonable request.
