# Supplementary figures and images for: Do We Need to Worry About Staphylococcus epidermidis ST0409KOC, a Cheese‐Isolated Strain With Bacteriocinogenic Properties?
Source: Mol Nutr Food Res. 2026 Jan 15;70(1):e70359. doi: 10.1002/mnfr.70359 (PMC12808867; doi:10.1002/mnfr.70359)

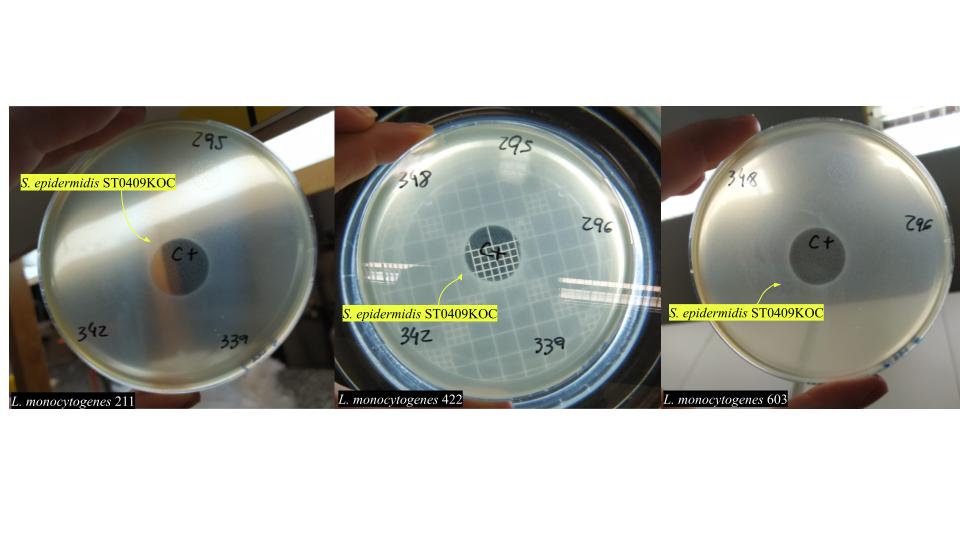

Supplement: Supplementary file 1 — Supplementary File: mnfr70359‐sup‐0001‐SuppMat.zip. [file MNFR-70-e70359-s001.zip › Supplementary material 1.jpg]

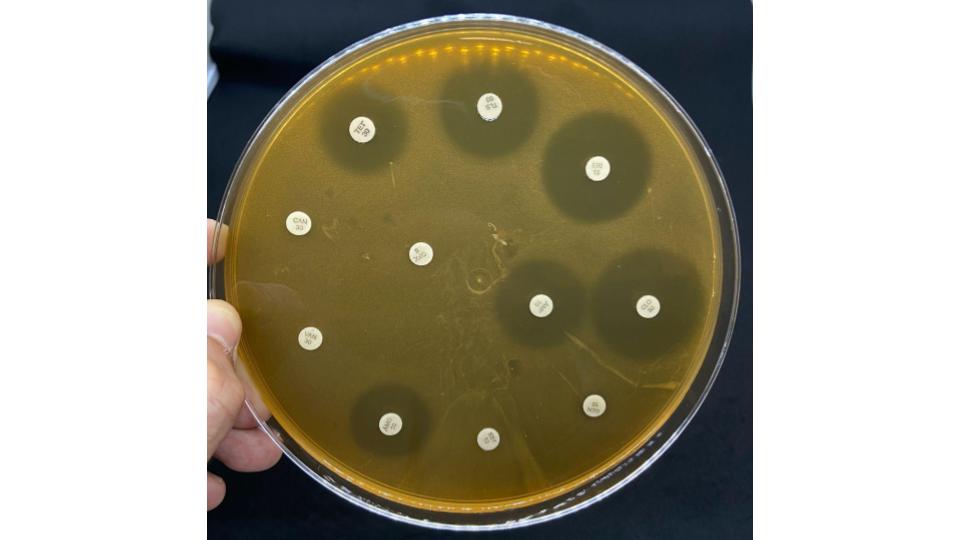

Supplement: Supplementary file 1 — Supplementary File: mnfr70359‐sup‐0001‐SuppMat.zip. [file MNFR-70-e70359-s001.zip › Supplementary material 2.jpg]

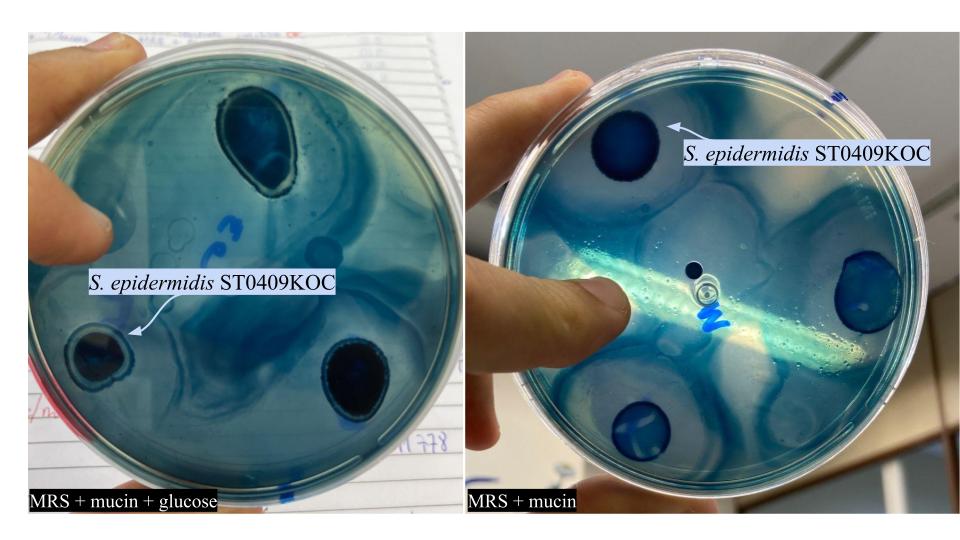

Supplement: Supplementary file 1 — Supplementary File: mnfr70359‐sup‐0001‐SuppMat.zip. [file MNFR-70-e70359-s001.zip › Supplementary material 3.jpg]

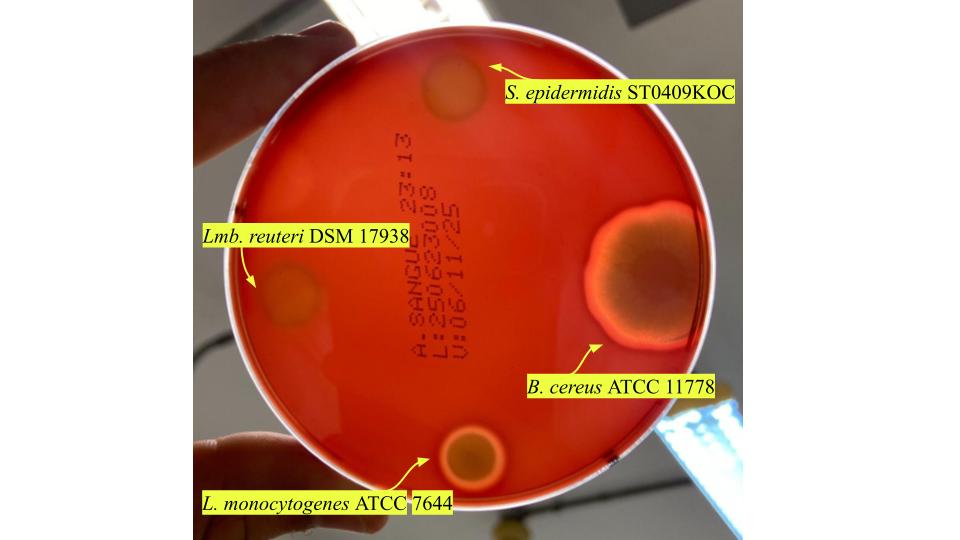

Supplement: Supplementary file 1 — Supplementary File: mnfr70359‐sup‐0001‐SuppMat.zip. [file MNFR-70-e70359-s001.zip › Supplementary material 4.jpg]

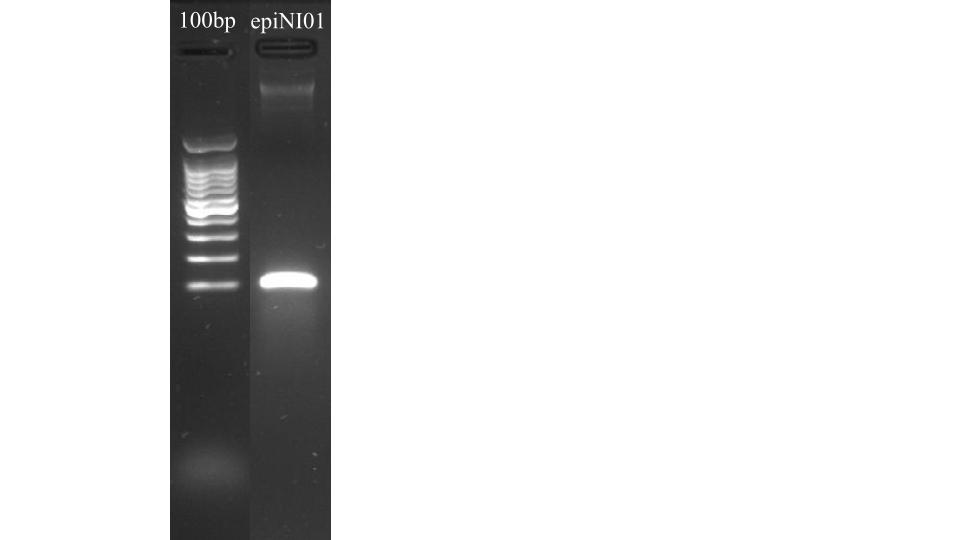

Supplement: Supplementary file 1 — Supplementary File: mnfr70359‐sup‐0001‐SuppMat.zip. [file MNFR-70-e70359-s001.zip › Supplementary material 5.jpg]
